# *Glycyrrhiza glabra* L. Extracts with Potential Antiproliferative and Anti-Migration Activities Against Breast and Gynecological Cancer Cell Lines

**DOI:** 10.3390/plants15030475

**Published:** 2026-02-03

**Authors:** Maria Rosaria Perri, Carmine Lupia, Máté Vágvölgyi, Attila Hunyadi, Sándor Bartha, Renáta Minorics, István Zupkó, Mariangela Marrelli, Filomena Conforti, Giancarlo Statti

**Affiliations:** 1 Department of Pharmacy, Health and Nutritional Sciences, University of Calabria, 87036 Rende, Italy; mariarosaria.perri@unical.it (M.R.P.); filomena.conforti@unical.it (F.C.); giancarlo.statti@unical.it (G.S.); 2 Mediterranean Ethnobotanical Conservatory, 88054 Sersale, Italy; studiolupiacarmine@libero.it; 3 National Ethnobotanical Conservatory, 85040 Castelluccio Superiore, Italy; 4 Institute of Pharmacognosy, University of Szeged, H-6720 Szeged, Hungary; vagvolgyi.mate@szte.hu (M.V.); hunyadi.attila@szte.hu (A.H.); 5 HUN-REN-SZTE Biologically Active Natural Products Research Group, H-6720 Szeged, Hungary; 6 Institute of Pharmacodynamics and Biopharmacy, University of Szeged, H-6720 Szeged, Hungary; bartha.sandor@stud.u-szeged.hu (S.B.); kanizsaine.minorics.renata@szte.hu (R.M.); zupko.istvan@szte.hu (I.Z.)

**Keywords:** *Glycyrrhiza glabra*, isoliquiritigenin, glycyrrhizin, 18β-glycyrrhetinic acid, HPLC, antiproliferative activity, anti-migration potential

## Abstract

*Glycyrrhiza glabra* L. (Fabaceae) is a plant species with already demonstrated countless biological properties and many more still to be discovered. Here, root sample extracts from different geographical areas were compared based on their phytochemical profiles and biological activities. Both raw and hydrolysate extracts, as well as 18β-glycyrrhetinic acid, glycyrrhizin, and isoliquiritigenin, considered as the main licorice secondary metabolites, were screened for antiproliferative and anti-migration properties in MCF-7, MDA-MB-231, A2780, HeLa, SiHa, and C33A breast and gynecological cancer cell lines. Hydrolysate extracts showed higher cytotoxicity than the raw extracts at the same final concentrations, 30 and 60 µg/mL, respectively. Among the standards, isoliquiritigenin showed the most pronounced cytotoxic activity, with inhibitory percentages exceeding 70% in each of the investigated cell lines at the lowest tested dose of 30 µg/mL. Then, the most effective extracts in the MTT assay, LIT2-H and LMO-H, were screened in a wound-healing test, demonstrating efficacy against ovarian (A2780) and cervical (C33A) cancer cell lines after 24 and 48 h of exposure.

## 1. Introduction

Cancer is considered the second leading cause of death worldwide, and it has been estimated that it will become the first by 2060, with more than 18 million deaths per year [1]. Breast cancer is the most commonly diagnosed type of cancer in women, representing a cluster of malignancies that can be classified into three main subtypes based on the presence and/or absence of estrogen (ER)/progesterone (PR) receptors and human epidermal growth factor (HER) 2. Luckily, the incidence of triple-negative breast cancer (absence of ER, PR, and HER2 receptors) is very low (15%) if compared to the hormone receptor-positive cancer subtype, which in 70% of cases is associated with a better prognosis [2]. Triple-negative cancer is the most aggressive type of breast cancer, commonly associated with resistance to therapy, low survival rate, and poor prognosis [3]. Gynecological cancers, a serious global health problem, account for almost 20% of total female malignancies, among which uterine cancer is the most common, followed by ovarian and cervical ones [4]. Among all reproductive cancers, ovarian cancer is considered one of the most fatal: it is usually diagnosed at the last stages, and there are no adequate screening programs. For a woman, the chance of developing ovarian cancer during her lifetime is 1 in 75, and it is estimated that the survival rate after 5 years is about 47%. Both benign and malignant ovarian cancers arise from epithelial, stromal, or germ cells, even though, in developed countries, about 90% have an epithelial origin [5,6,7]. Although there are no specific symptoms for ovarian cancer, some signs, such as abdominal bloating and pain, modified bowel habits, frequency of urinating, and feeling of early satiety, should not be underestimated [6]. Cervical cancer is the second most common cancer among women: Global Oncology 2020 assessed around 57,000 diagnoses per year [8]. In 2018, the World Health Organization (WHO) established a plan to eliminate cervical cancer globally [9]. This cancer is also known as the “disease of disparity”, because of the difference between incidence and mortality rate between developed and non-developed countries. High-income countries, in fact, have made widespread human papillomavirus (HPV) screening programs and vaccination campaigns available [10,11,12]. HPV infection, together with progression to precancer lesion and subsequent invasion, is a multistep process involved in cervical cancer carcinogenesis [13]. A small portion of people affected by cervical cancer test negative during the HPV test. HPV-negative cervical cancer, in fact, seems to represent a separate cluster of malignancies, associated with poor prognosis: it is not commonly diagnosed at an early stage, is predisposed to metastasis, and has a minor overall and disease-free survival rate [14]. Conventional cancer treatments such as chemotherapy and radiotherapy are effective; on the other hand, they present many severe and non-negligible side effects. Currently, there is increasing attention to and interest in the discovery of new plant-based medicines. Moreover, the chemoprevention research area is gaining even more attention and accreditation as a promising medical approach able to prevent, reduce, or reverse the tumorigenesis process. The strategy concerns the use of synthetic or, in most cases, natural dietary-origin products aimed at targeting the tumorigenesis process at different levels: primary, secondary, or tertiary. Primary chemoprevention is about preventing the formation of premalignant lesions; secondary chemoprevention slows the progression of precancerous lesions into cancerous ones; and tertiary chemoprevention blocks the spread of primary cancer [15,16]. *Glycyrrhiza glabra* L., commonly known as licorice, is a very popular traditional medicine belonging to the Fabaceae (Leguminosae) family, widely distributed in the Mediterranean region as well as in India, Russia, and China. The term *Glycyrrhiza* dates back to ancient Greece, and it means “sweet root” [17]. It is a perennial herbaceous plant that grows along rivers and streams in fertile sands. Although licorice is commonly known for its application in foods and beverages, its rhizomes and roots, the most interesting parts of licorice were traditionally used for the treatment of digestive disorders, including ulcer, hyperdipsia, colic, flatulence, and respiratory tract disease such as cough, asthma, and sore throat. Moreover, the use of licorice, alone or in combination with other herbal drugs, has been reported in the treatment of psoriasis, prostate cancer, malaria, etc. [18]. The main bioactive constituents of *G. glabra* are triterpenes, saponins, flavonoids, coumarins, and phenolics. Glycyrrhizin, a triterpene saponin, is the most abundant active principle of licorice roots, representing almost 10–25% of the extracts and being responsible for the plant’s sweet taste [19,20]. The anti-inflammatory, antidepressant, antidiabetic, and antiulcer activities of this species have already been demonstrated [21]. Inevitably, herbal drugs, after ingestion and before absorption, come into contact with intestinal microflora that metabolize compounds: after oral administration and through the activity of intestinal bacteria, glycyrrhizin is hydrolyzed to 18-glycyrrhetinic acid, as the main product, and to 18β-glycyrrhetinic acid-3-O-β-D-glucuronide, as a minor one [22]. An in vivo study demonstrated that hydrolysis by intestinal bacteria is fundamental for 18β-glycyrrhetinic acid absorption after 17 h of oral administration in rats [23]. Glycyrrhizin and overall licorice extracts’ toxicity were reviewed by Nazari and colleagues (2017) and categorized into acute, sub-acute, sub-chronic, and chronic states. Acute analyses, revealing the toxicity promoted by a single dose exposure within 14 days, were investigated, demonstrating that both aqueous and ethanol *G. glabra* root extracts did not show mortality after a 1000 mg/kg single oral dose in female albino rats, even if behavioral assessment, including alertness, loco-motor activity, and reactivity to touch, decreased within 3 h [24]. Another study conducted in Swiss male and female albino mice confirmed that 1500 mg/kg of *G. glabra* root extract remained safe, although physical changes were observed [25]. The acute toxicity of glycyrrhizin, instead, may vary depending on the salt type and the administration form: toxicity levels were assessed at 412 mg/kg for potassium glycyrrhizinate administered intravenously and at 12,700 mg/kg for ammonium glycyrrhizate administered orally [26]. This can partially be explained because the oral bioavailability of glycyrrhizin in the extracts was decreased by licorice bioactive compounds, which interfere with intestinal absorption [27]. Sub-acute toxicity, determined through different administrations between 14–28 days, revealed that both aqueous *G. glabra* root extract (100, 250, and 500 mg/kg) and glycyrrhizin inhibited the adrenal–pituitary axis and the extract also induced hypermineralocorticoidism [28]. No-Observed Adverse Effect Level (NOAEL) for water licorice extract was greater than 200 mg/kg in a sub-chronic exposure study, with no toxicity to reproductive organs. On the other hand, glycyrrhizin (0.1–1 mg/mL) administered in water showed significant mineralcorticoid syndrome, including hypertension, hypokalemia, and hypernatremia in Sprague Dawley rats. Chronic toxicity was assessed at 4 mg/L of *G. glabra* root extract for 45 days in male black molly fish. Also, caution is needed when administering the extract during pregnancy [29]. Cytotoxicity properties were determined using a various sources of both in vitro and in vivo evidence, demonstrating that licorice mixed extracts (obtained by combining plant crude extracts and isolated active principles) exert anticancer properties by inhibiting cell viability, inducing cell cycle arrest, apoptosis, and autophagy, and suppressing metastasis. Moreover, the association between chemotherapeutics and licorice compounds enhanced the drugs’ anticancer effects and, in turn, reduced their side effects. The trichlorometane, hexane, ethyl acetate, and 70% methanol licorice extracts significantly affected MCF-7 cell viability in a dose- and time-dependent manner, while the 70% ethanol licorice extract inhibited MDA-MB-231 cell viability [30]. As regards the pure single compounds, if glycyrrhizic acid (100 µM) showed slight or no cytotoxicity in human cervical cancer cells, glycyrrhetinic acid exerted significant inhibitory activity in SiHa cell lines [31]. Isoliquiritigenin, instead, was the most effective against human ovarian cancer in vitro models (OVCAR-5 and ES-2), inducing cytotoxicity in a dose- and time-dependent manner, together with apoptosis and autophagy [32].

The idea and innovation behind this work lie primarily in the hydrolysis undergone by the extracts. This process, in fact, not only converts glycyrrhizin into its metabolites, as previously discussed, but also has the potential to reduce all of its associated side effects, including pseudoaldosteronism, high blood pressure, hypokalemia, and hypernatremia. Moreover, the hydrolysis process, in this case, is not selective, affecting all other eligible compounds. To the best of our knowledge, this is the first study to evaluate and compare the effects of raw and hydrolyzed *G. glabra* extracts on potential antiproliferative and anti-migratory properties using the MTT assay and wound-healing test, respectively. Based on this evidence, this work aimed to gain further insights into the antitumor properties of licorice, with particular focus on its bioactive compounds.

## 2. Results and Discussion

### 2.1. Phytochemical Analyses

**Figure 1:**
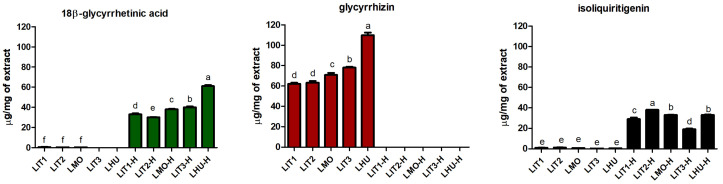
Graphical representation of 18-β glycyrrhetinic acid, glycyrrhizin, and isoliquiritigenin HPLC analyses in both raw and hydrolysate *G. glabra* L. extracts expressed as µg/mg of extract of 18-β glycyrrhetinic acid, glycyrrhizin, and isoliquiritigenin. Different letters among columns indicate statistically significant differences at *p* < 0.05 (Bonferroni post hoc test). Data for Italian and Moroccan specimens were derived from [33]. Sample abbreviation codes are reported in Table 1.

The 18β-glycyrrhetinic acid (18β), glycyrrhizin (GLY), and isoliquiritigenin (ISL) content in some licorice specimens was previously investigated [33]. Here, these extracts were compared with a sample collected in another geographical area: Szeged, Hungary (LHU, LHU-H). All samples were analyzed simultaneously and uniformly under the same analytical conditions (Figure 1). LHU-H possessed the highest 18β content (61.00 ± 1.01 µg/mg of extract), followed by LIT3-H and LMO-H. As regards GLY, as expected, it was found only in the raw extracts, not in the hydrolysate ones. GLY, in fact, is a triterpenoid saponin that has no oral bioavailability. Through the action of intestinal bacteria, it undergoes hydrolysis, transforming it into the aglycone form, glycyrrhetinic acid [34]. Also, in this case, the highest value of the standard was observed in the LHU sample (109.84 ± 2.64 µg/mg of extracts), while intermediate values were observed in the LIT3 and LMO extracts. ISL was detected in all of the investigated samples, both raw and hydrolysates. Still, in this case, the highest amount was possessed by LIT2-H (38.00 ± 0.04 µg/mg of extract), followed by LMO-H and LHU-H, which showed comparable amounts with no statistical difference using one-way ANOVA with the Bonferroni post hoc test, *p* < 0.05. LHU and LHU-H extracts’ HPLC chromatograms are reported in Figures S1 and S2. Cultivation area significantly affects agro-morphological and phytochemical traits of *G. glabra*. Eghlima and colleagues (2025) found differences in the phytochemical composition, including glabridin, glycyrrhizic acid, and liquiritigenin, and in the total phenolic content of plants belonging to Rayen, Eghlid, Kalat, and Zanjan [35]. Another study conducted by Astaf’eva and colleagues (2014) [36] compared the amount of glycyrrhizin and 18β-glycyrrhetinic acid in two 50% ethanolic extracts from Astrakhan (Russia) and Calabria (Italy), revealing that both of the investigated compounds were significantly higher in the sample from Russia, suggesting that these findings could be due to climatic and geographic characteristics. Moreover, the content of these compounds was directly proportional to the extracts’ antibacterial activity [36].

### 2.2. Antiproliferative Assay

**Figure 2:**
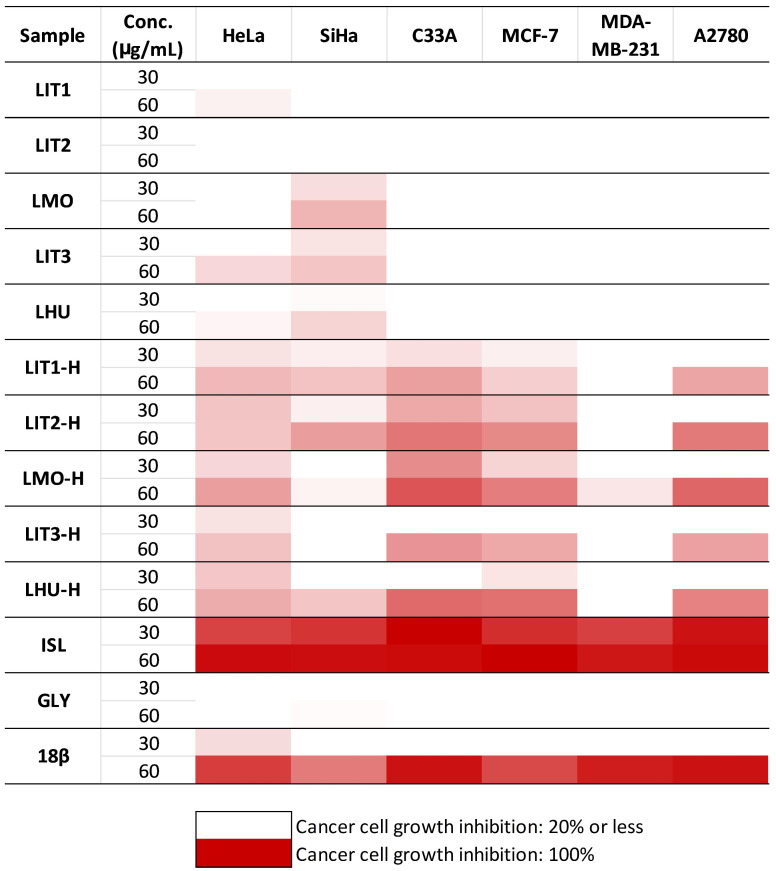
Antiproliferative properties of the investigated raw and hydrolysate *G. glabra* extracts and isoliquiritigenin (ISL), glycyrrhizin (GLY), and 18β-glycyrrhetinic acid (18β), pure main compounds. The exact value percentages are given in Table S1.

In this project, we evaluated the in vitro antiproliferative activity of five *G. glabra* extracts derived from different geographical areas and their hydrolysates on a panel of adherent human gynecological cancer cell lines. The extracts were tested against breast (MCF-7 and MDA-MB-231), ovarian (A2780), and cervical (HeLa, SiHa, and C33A) carcinoma cell lines. Concerning the antiproliferative effects of the prepared crude extracts of *G. glabra*, LIT1, LMO, LIT3, and LHU exerted some modest inhibitions against the two papillomavirus-related cervical cell lines HeLa and SiHa only. The hydrolysate extracts of *G. glabra* (LIT1-H, LIT2-H, LMO-H, LIT3-H, and LHU-H) displayed a more pronounced inhibitory effect on cell proliferation in the tested cancer cell lines than their corresponding original pairs (Figure 2). They demonstrated the highest activity against C33A cervical cancer cells, followed by MCF-7, A2780, and HeLa cells. If we compare the cell growth-inhibitory values of corresponding pairs of extracts on SiHa cells, inconsistent alterations are observed. LIT1-H and LIT2-H extracts displayed stronger antiproliferative activity on SiHa cells than their non-hydrolysate extract pairs. However, LHU and LHU-H extracts showed similar cell growth-inhibitory effects at both applied concentrations. Finally, the original extracts, LMO and LIT3, evoked a more substantial inhibitory effect on SiHa cells than their hydrolysate variants (Figure 2, Table S1).

Three pure compounds, ISL, GLY, and 18β, were also investigated, since they are the main components of *G. glabra* and have already been shown to possess a cell-proliferation-inhibiting effect on different cancer cell lines [37,38,39]. ISL demonstrated the strongest antiproliferative capacity in our experiments (Figure 2, Table S1). It displayed an inhibitory effect on cell proliferation of over 79% across all tested gynecological cancer cell lines, even at the lower concentration (30 µg/mL). Moreover, 18β exhibited substantially less pronounced antiproliferative activity, resulting in considerable (i.e., 60–95%) cancer cell growth inhibition only at 60 µg/mL, and the lower concentration was practically ineffective. The antiproliferative activity of GLY was negligible; the obtained inhibition values were below 25% even at 60 µg/mL.

Regarding the mechanism underlying the observed cell-growth-inhibitory activity, elucidating its molecular mechanism is beyond the scope of our current work. However, it seems reasonable that the effect can be attributed to different classes of compounds. Licorice flavonoids are well-known natural products with anticancer properties that act through multiple signaling pathways, including MAPK, PI3K/AKT, and NF-κBs [40]. The plant chalcones elicit mitochondrial apoptosis, while High Mobility Group Box 1 (HMGB1), a cytokine, has been identified as a molecular target for glycyrrhizin [41,42]. In a study by Farooqui and colleagues (2018), the antiproliferative and antiapoptotic properties of glycyrrhizin were investigated in human cervical cancer HeLa cells. In their study, 24 h exposure to glycyrrhizin (20, 40, 80, 160, and 320 µM) significantly affected cell viability at percentages of 24.63%, 47.40%, 54.48%, 67.46%, and 88.43%, respectively, increasing nuclear condensation, DNA fragmentation, and the production of Reactive Oxygen Species (ROS) [43]. Ahmad and colleagues (2022) investigated the biological properties of *G. glabra* extracts from different geographical origins. The Indian samples, tested at concentrations ranging from 1 to 500 µg/mL, showed antiproliferative activity with an IC_50_ of 56.10 ± 2.38 µg/mL in the MCF-7 cell line after 48 h [44]. Wang and coworkers (2020) reviewed the potential antitumor effects of isoliquiritigenin in breast, colon, gastrointestinal, lung, ovarian cancers, leukemia, and melanoma, both in vitro and in vivo. In vivo studies concerning breast cancer highlighted that isoliquiritigenin inhibited cancer growth and angiogenesis in MDA-MB-231-bearing female nude mice at doses of 20 and 50 mg/kg/day, while oral administration in MDA-MB-231-bearing Balb/c nu/nu mice at 10 and 20 mg/kg/day, 5 times/week, also induced antimetastatic properties. An additional in vivo study showed that isoliquiritigenin, at subcytotoxic doses (12 and 25 mg/kg), was effective in inhibiting ovarian cancer by antagonizing epithelial-to-mesenchymal transition (EMT) through modulation of the Transforming Growth Factor (TGF) signaling pathway in 6-week-old female athymic nude mice [45]. In general, both *G. glabra* extracts and licorice phytochemicals (e.g., 18β-glycyrrhetinic acid and glycyrrhizic acid) have already demonstrated potential anticancer properties, as shown by different in vitro and in vivo studies. Concentrations ranging from 0 to 12.5, 25, 50, and 100 µg/mL of a licorice methanolic extract showed potential anticancer activity against the intestinal carcinoma cell line (Caco-2) and the prostate carcinoma cell line (PC-3) [46]. *Glycyrrhiza* ethanolic extracts also showed antiproliferative properties in MCF-7 cancer cells, in an adipose-dependent manner. A licorice extract (70% methanol) showed efficacy in human monoblastic leukemia U937 cells by triggering apoptosis [47]. Also, the other licorice compounds already showed significant antiproliferative activity. Ahmad and coworkers (2022) investigated the potential antiproliferative and apoptotic effects of glycyrrhizin in HPV16+ CaSki CCa cells; as a result, the molecule inhibited cell proliferation and increased apoptosis by enhancing ROS production, DNA fragmentation, and disrupting mitochondrial membrane potential [48]. Another study conducted in 2020 highlighted the effects of glycyrrhizin in HPV-negative C33A cervical cancer cells. Data showed that glycyrrhizin induced cytotoxicity, apoptosis, and arrested the cell cycle at the G0/G1 phase, associated with decreased cyclin D1 expression through downregulation of the Notch pathway [49]. 18β-glycyrrhetinic acid induced cell cycle arrest at the G0/G1 phase and apoptosis in HPV18+ HeLa cervical cancer cells, significantly inhibiting cell proliferation in a dose- and time-dependent manner [50].

### 2.3. Wound-Healing Assay

**Figure 3:**
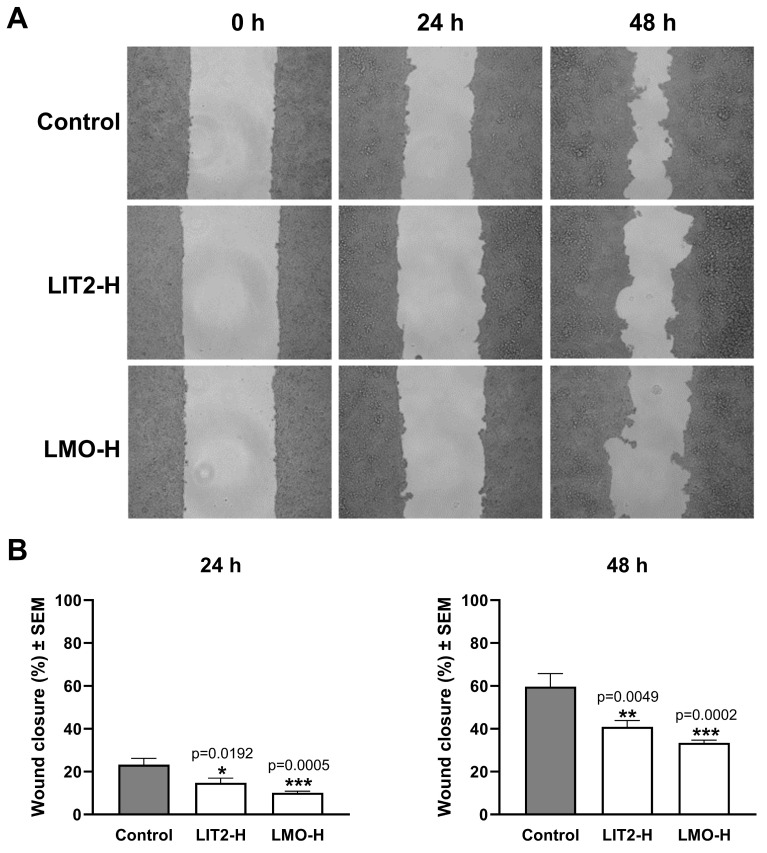
(**A**) Effect of two hydrolysate extracts (LIT2-H and LMO-H) of *G. glabra* on cancer cell migration investigated by means of wound healing assay on ovarian (A2780) cancer cell lines. (**B**) Cell migration inhibitory effect of LIT2-H and LMO-H on A2780 cells. Data were derived from two independent experiments, each with four replicates. *, **, and *** indicate significance at *p* < 0.05, *p* < 0.01, and *p* < 0.001, respectively, compared to the control (Dunnett’s multiple-comparison test).

**Figure 4:**
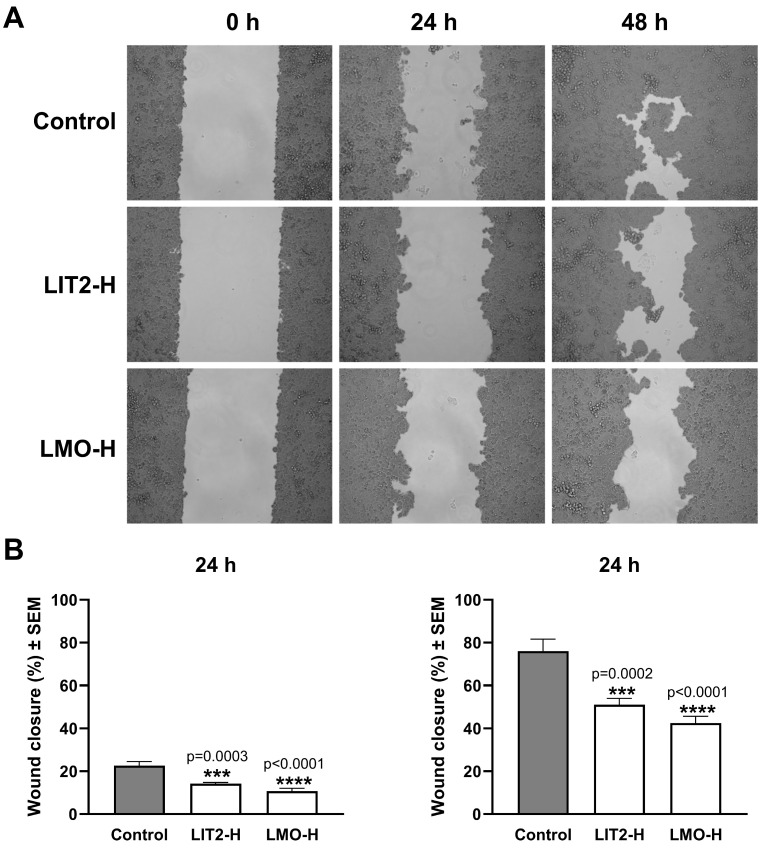
(**A**) Effect of two hydrolysate extracts (LIT2-H and LMO-H) of *G. glabra* on cancer cell migration investigated by means of wound-healing assay on cervical (C33A) cancer cell lines. (**B**) Cell migration inhibitory effect of LIT2-H and LMO-H on C33A cells. Data were derived from two independent experiments, each with four replicates. *** and **** indicate significance at *p* < 0.001 and <0.0001, respectively, compared to the control (Dunnett’s multiple-comparison test).

The wound-healing assay is suitable for demonstrating the inhibitory activity of the tested extracts on cancer cell migration, an essential component of their antimetastatic characteristics. Two hydrolysate extracts with high antiproliferative activity, LIT2-H and LMO-H, were investigated in wound-healing assays. Their inhibitory effects on cell migration were demonstrated on A2780 and C33A cell lines. Both extracts significantly inhibited wound closure in both investigated cell lines at 10 μg/mL compared to untreated control cells (Figure 3 and Figure 4). Moreover, their significant inhibitory effects were already evident after 24 h of incubation. Similar to cell division, cell migration is a complex and incompletely understood process; therefore, potential targets for pharmacological intervention are not fully described. Flavonoids are widely accepted as inhibitors of epithelial–mesenchymal transition, a key event in metastasis formation, by modulating a diverse array of endogenous regulatory proteins, including integrins, matrix metalloproteinases, and several kinases [51].

In this study, the best biological activity, in terms of antiproliferative and anti-migratory effects, is attributed to two samples, LIT2-H and LMO-H hydrolysate extracts, both of which possess the highest content of isoliquiritigenin, as highlighted by HPLC analyses [33]. Additionally, the MTT assay performed on individual standards showed that isoliquiritigenin had cytotoxicity greater than 70% even at the lowest tested concentration (30 μg/mL). The anti-invasive and anti-migratory properties of some licorice phytochemicals, including 18β-glycyrrhetinic acid, isoliquiritigenin, and licoricidin, were investigated in gastric cancer cell lines. Of note, 18β-glycyrrhetinic acid significantly decreased levels of vimentin, Matrix Metalloproteinase-2 (MMP-2), and MMP-9, and increased levels of E-cadherin, all factors involved in the metastasis and progression process, in SGC-7901 gastric cancer cells. Isoliquiritigenin and licoridicin inhibited epithelial–mesenchymal transition (EMT)-associated proteins and the P13K/AKT/mTOR pathway in both MGC-803 and MKN28 human gastric cancer cells [52].

## 3. Materials and Methods

### 3.1. Reagents

For our experiment, 18β-glycyrrhetinic acid, glycyrrhizin, and isoliquiritigenin, analytical standards suitable for HPLC, were purchased from Sigma-Aldrich S.p.a. (Milan, Italy). All of the reagents and solvents (HPLC-grade and reagent-grade) were purchased from Sigma-Aldrich (Merck KGaA, Darmstadt, Germany).

### 3.2. Plant Material Extraction and Hydrolysis Process

The investigated *G. glabra* samples belonged to different geographical areas. The collected samples were from Montalto Uffugo, Calabria, Italy (a voucher specimen was deposited at the Mediterranean Ethnobotanical Conservatory, Sersale, Catanzaro, position number 37 of the Fabaceae section). In contrast, the other samples from Rossano (Calabria, Italy), Morocco, and Szeged (Hungary) were commercially available in powder form (Table 1). The collected samples were allowed to dry at room temperature and then properly pulverized through a disintegrator. All of the shredded samples were passed through a 600 µm sieve, extracted with 80% MeOH by means of ultrasonication for 20 min, and dried under nitrogen. Moreover, part of the licorice powder was also subjected to a hydrolysis process: 100 mg of powder was extracted with 5 mL MeOH for 10 min by means of ultrasonication, and the obtained extract, properly filtered, was mixed with 5 mL of HCl and incubated in a water bath (100 °C) for 60 min. The mixture was then extracted with 20 mL of ethyl acetate, and a NaCl solution was added to the organic phase to remove the acid. Na_2_SO_4_ was added to the obtained organic phase and then filtered and dried using a rotary evaporator [33].

**Table 1:** *G. glabra* abbreviation codes, geographical origins.

Code	Geographical Origin
LIT1 (raw) LIT1-H (hydrolysate)	Italy
LIT2 (raw) LIT2-H (hydrolysate)	Italy
LMO (raw) LMO-H (hydrolysate)	Morocco
LIT3 (raw) LIT3-H (hydrolysate)	Italy
LHU (raw) LHU-H (hydrolysate)	Hungary

### 3.3. HPLC Analyses

*G. glabra* raw and hydrolysate extracts were investigated through a Kinetex XB-C18 250 × 4.6 mm 5 µm column (Phenomenex Inc., Torrance, CA, USA). The following gradient separation was applied by using water with 0.1% formic acid as solvent A and acetonitrile with 0.1% formic acid as solvent B: 25–45% B *v*/*v*, gradually increasing from 0 to 30 min; 45–100% B *v*/*v*, gradually increasing from 30 to 60 min [33,53]. The mobile phase flow rate was 1 mL/min. The RP-HPLC investigations were carried out on a dual-pump (PU-2080) Jasco HPLC instrument (Jasco International Co., Ltd., Hachioji, Tokyo, Japan) equipped with an MD-2010 Plus PDA detector operating at 255 nm, 370 nm, and 210–410 nm to record the maximum absorbance chromatogram. All measurements were carried out at 25 °C. In each extract, the presence of three main licorice components, 18β-glycyrrhetinic acid, glycyrrhizin, and isoliquiritigenin, was investigated and subsequently quantified. For each standard, an eight-point calibration curve was recorded (concentrations ranging from 0.0001 to 1 mg/mL in HPLC-grade methanol) to determine the linear range. Linearity of the method was assessed through R^2^ evaluation (Table 2). The HPLC chromatograms for the 18β, GLY, and ISL standards are reported in Figure S3.

**Table 2:** Linear range, standard calibration curve, and R^2^ assessment of 18β-glycyrrhetinic acid, isoliquiritigenin, and glycyrrhizin.

Reference Compounds	Linear Range	Standard Calibration Curve	R^2^
18β-glycyrrhetinic acid (18β)	1–0.001 mg/mL	y = 10,000,000x + 45,832	0.9998
glycyrrhizin (GLY)	1–0.05 mg/mL	y = 7,000,000x – 285,127	0.9995
Isoliquiritigenin (ISL)	0.5–0.0001 mg/mL	y = 50,000,000x + 132,335	0.9994

Results are expressed as mean ± S.D. (n = 3).

### 3.4. Antiproliferative Assay

Growth inhibitory effects of the present extracts and their reference compounds were characterized using standard MTT (3-(4,5-dimethylthiazol-2-yl)-2,5-diphenyltetrazolium bromide) assay on a panel of adherent human cancer cell lines of gynecological origin. The extracts of interest were tested against cervical (HeLa, SiHa, and C33A), breast (MDA-MB-231 and MCF-7), and ovarian (A2780) carcinoma cell lines. Cells were obtained from the European Collection of Authenticated Cell Cultures (ECACC, Salisbury, UK), except for SiHa and C33A, which were purchased from the American Tissue Culture Collection (ATCC, Manassas, VA, USA). Cells were cultured in minimal essential medium (EMEM) supplemented with 10% fetal bovine serum (FBS), 1% non-essential amino acid (NEAA) mixture, and 1% streptomycin–amphotericin B mixture. Culture media were purchased from Capricorn Scientific Ltd. (Ebsdorfergrund, Germany), while the supplements were obtained from Lonza Group Ltd. (Basel, Switzerland). All lines were seeded at 5000 cells/well, except C33A, which was seeded at 10,000 cells/well. Cells were then treated with all extracts and reference compounds at two concentrations (30 µg/mL and 60 µg/mL) [54] and incubated for 72 h at 37 °C. The maximal final solvent percentage (DMSO) was 0.6%. After incubation, 20 µL of 5 mg/mL MTT solution was added to the wells. After a 4 h incubation period, the supernatant was removed, and the converted formazan crystals were solubilized with 100 µL/well DMSO for 60 min at 37 °C with shaking. The samples were assayed at 545 nm using a microplate reader (BMG Labtech, Ortenberg, Germany).

### 3.5. Wound-Healing Assay

C33A and A2780 cell suspensions were prepared in EMEM containing 2% FBS, 1% NEAA mixture, and 1% streptomycin–amphotericin B mixture and then seeded onto 12-well microplates fitted with special silicone inserts (Ibidi GmbH, Gräfeling, Germany) at a density of 100,000 cells/insert. Microplates containing A2780 cells were incubated for 24 h, and those containing C33A cells were incubated for 48 h at 37 °C to allow the cells to attach to the bottom and reach (near) 100% confluency. After the incubation period, the inserts were gently removed from the wells, and the cells were washed with serum-free EMEM and treated with the tested extracts (LIT2-H or LMO-H at 10 µg/mL). The anti-migratory effect was characterized by determining the extent of cell-free areas in treated and untreated control samples using ImageJ software (version 1.53k), based on images taken with a microscope (NIKON Eclipse TS100; Nikon Instruments Europe B.V., Amstelveen, The Netherlands) fitted with a camera at 0, 24, and 48 h.

### 3.6. Statistical Analyses

HPLC analyses were performed in triplicate, and data are expressed as mean ± S.D. (n = 3). MTT mean values were obtained from two independent measurements, each assessed in five parallel wells. Wound-healing data were obtained from two independent measurements, each with four replicates. Statistical significance among samples was assessed using one-way ANOVA with the Bonferroni post hoc test (HPLC), Tukey’s test (MTT assay), and Dunnett’s multiple comparison test (wound-healing assay).

## 4. Conclusions

*G. glabra* extracts and the secondary metabolites they produce have already shown a plethora of interesting biological activities that have attracted, over the years, the attention of the scientific community. Here, the biological activity of extracts coming from different geographical areas was analyzed and compared. Antiproliferative and anti-migratory assays indicated that hydrolysis significantly increased the biological activity of *G. glabra* extracts, thereby ameliorating their effects. This process, even if only partially, can serve as a starting point for better elucidating the mechanisms by which *G. glabra* extracts act within the human body. Samples with the best activity showed the highest content of isoliquiritigenin in HPLC analyses, suggesting that this molecule could be directly involved in the cytotoxicity and anti-migration potential of the extracts. In addition, licorice bioactive compounds such as isoliquiritigenin and glycyrrhizin/glycyrrhetinic acid are currently under analysis because of their potential to mitigate chemotherapeutic side effects when administered in combination with anticancer drugs [55]. All of these findings suggest that the *G. glabra* species, although widely studied worldwide, still has untapped potential that deserves further investigation, presumably using more complex experimental systems, including in vivo tumor models.

## Data Availability

Data are contained within the article.

## Supplementary Materials

The following supporting information can be downloaded at https://www.mdpi.com/article/10.3390/plants15030475/s1. Figure S1: RP-HPLC chromatogram of the LHU sample; Figure S2: RP-HPLC chromatogram of the LHU-H sample; Figure S3: 18β, GLY and ISL standards HPLC chromatograms; Table S1: Antiproliferative properties of the investigated five extracts of *Glycyrrhiza glabra* L., their hydrolyzed variants, and pure main components.

## Abbreviations

The following abbreviations are used in this manuscript:

EMEM	Minimal Essential Medium
EMT	Epithelial–Mesenchymal Transition
ER	Estrogen
FBS	Fetal Bovine Serum
HER 2	Human Epidermal Growth Factor 2
HMGB1	High Mobility Group Box 1
HPLC	High-Performance Liquid Chromatography
HPV	Human Papillomavirus
MMP	Matrix Metalloproteinase
NEAA	Non-Essential Amino Acids
NOAEL	No-Observed Adverse Effect Level
PR	Progesterone
TGF	Transforming Growth Factor
ROS	Reactive Oxygen Species
WHO	World Health Organization
