# Supplementary material for: Glycyrrhiza glabra L. Extracts with Potential Antiproliferative and Anti-Migration Activities Against Breast and Gynecological Cancer Cell Lines
Source: Plants (Basel). 2026 Feb 3;15(3):475. doi: 10.3390/plants15030475 (PMC12899981; doi:10.3390/plants15030475)
Supplement: Supplementary file 1 [file plants-15-00475-s001.zip › plants-4086780-supplementary.pdf]

Figure S1: RP-HPLC chromatogram of LHU sample

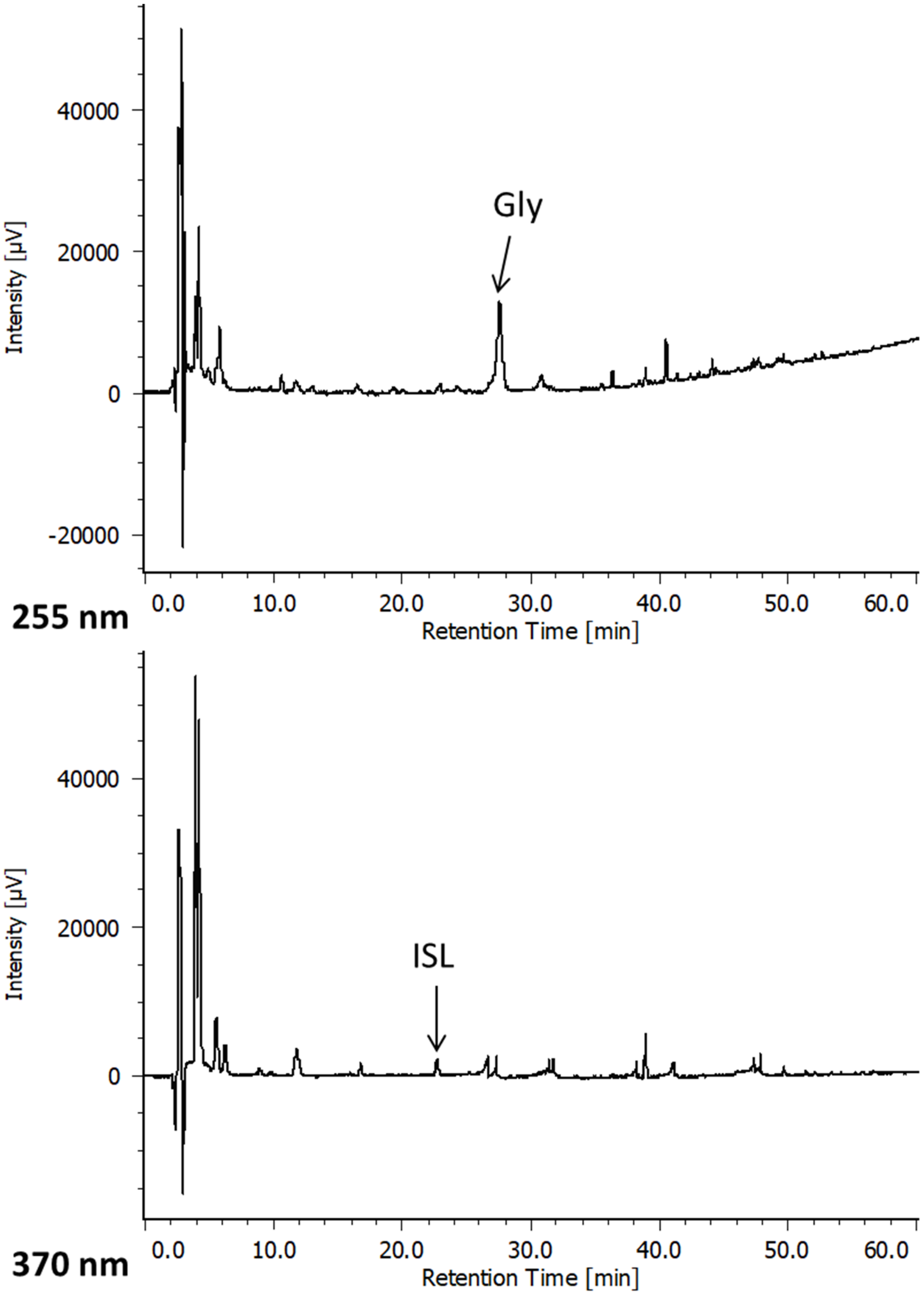

Chromatograms recorded at 255 nm and 370 nm, respectively. Codenames used are as follows. GLY: glycyrrhizin; ISL: isoliquiritigenin.

Figure S2: RP-HPLC chromatogram of LHU-H sample

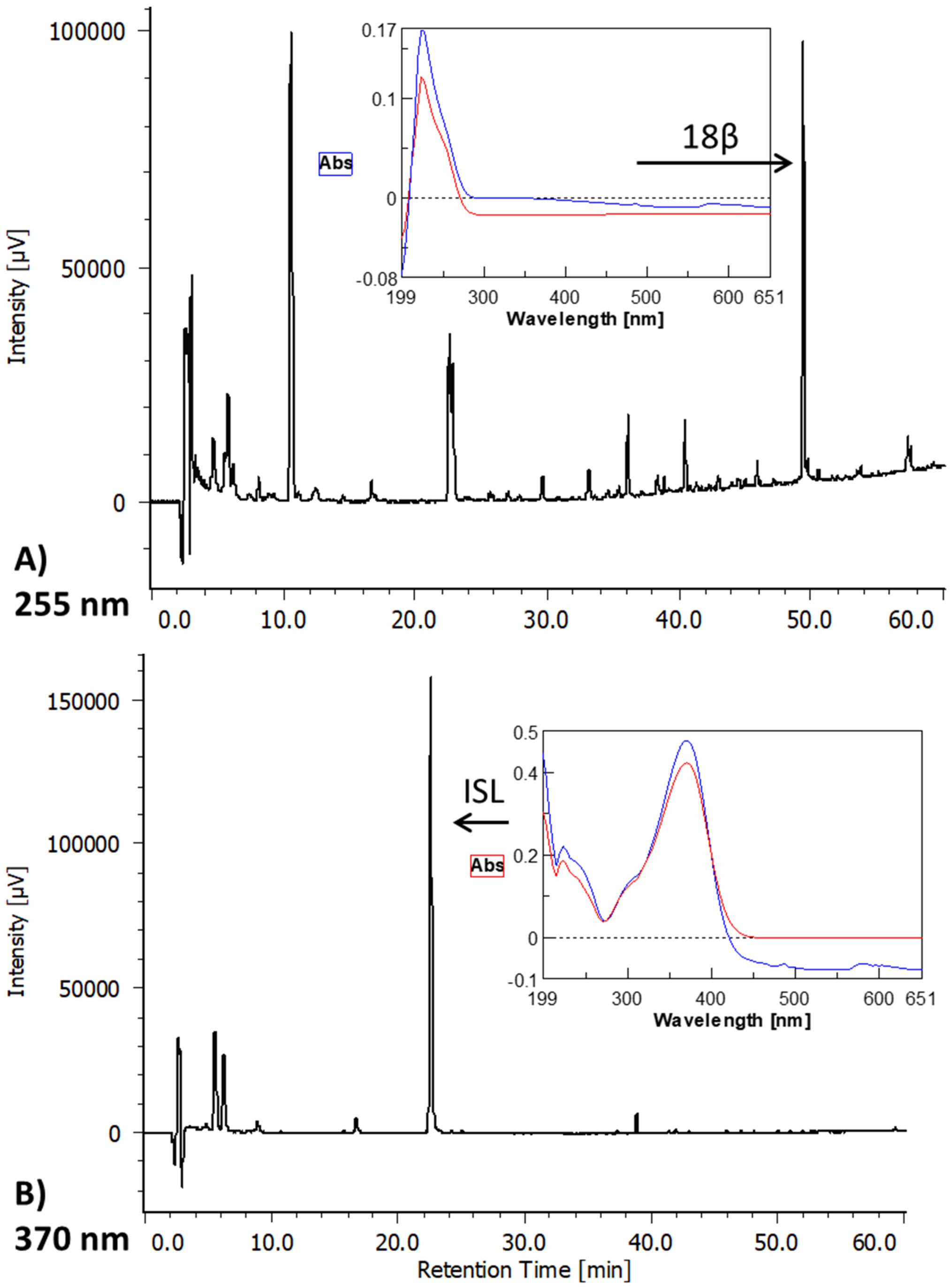

Chromatograms recorded at 255 nm and 370 nm, respectively. Codenames used are as follows. 18β: 18β-glycyrrhetinic acid; ISL: isoliquiritigenin. UV-VIS absorbance spectra of analytes 18β and ISL in native samples were indicated in overlap with their corresponding standard (blue line: standard, red line: analyte in native sample).

Figure S3: 18β, GLY and ISL standards HPLC chromatograms.

18β-glycyrrhetinic acid – 255 nm

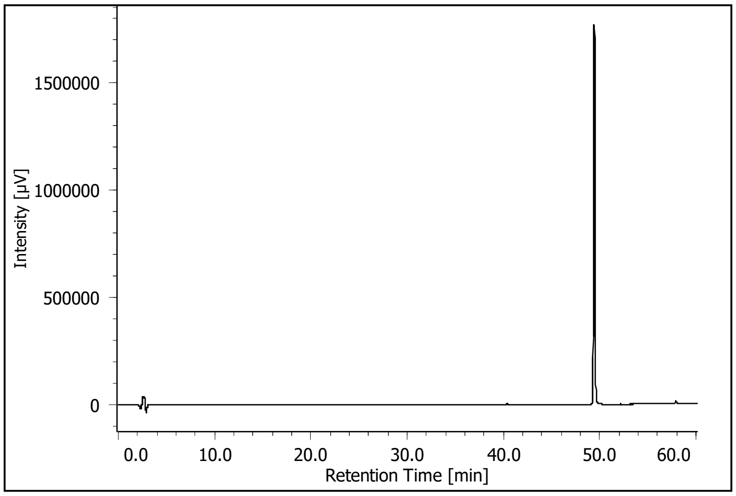

Isoliquiritigenin – 370 nm

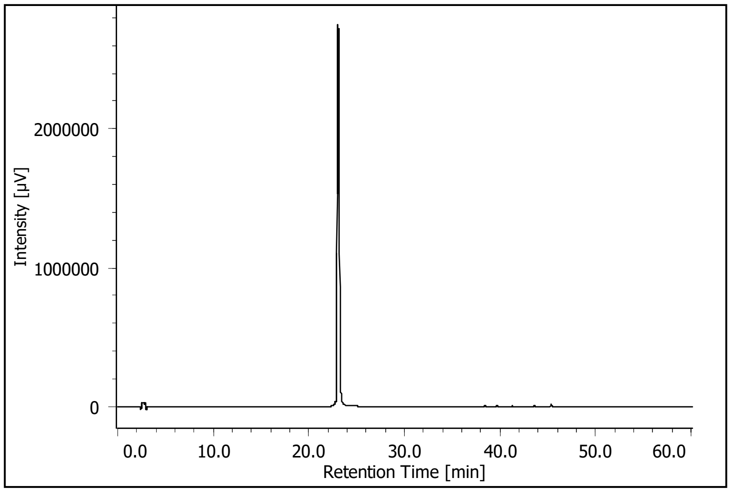

Glycyrrhizin – 255 nm

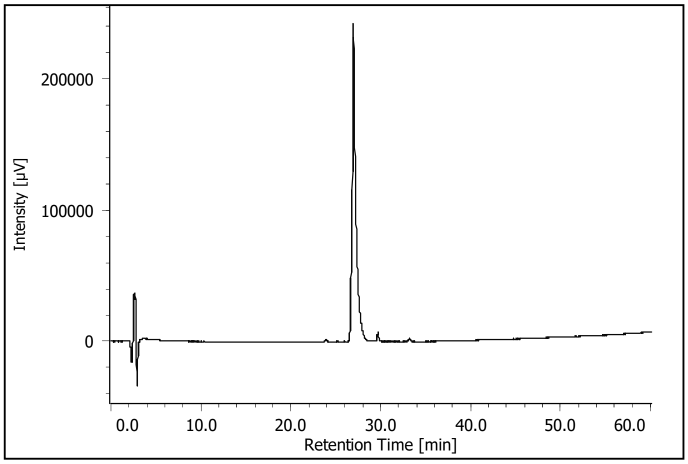

Table S1. Antiproliferative properties of the investigated five extracts of *Glycyrrhiza glabra* L., their hydrolyzed variants and pure main components.

Samples	Conc. [μg/mL]	Cell growth inhibition (%) ± S.E.M[a]					
		HeLa	SiHa	C33A	MCF-7	MDA-MB-231	A2780
LIT1	30	< 20	< 20	< 20	< 20	< 20	< 20
	60	24.62 ± 3.5[d,e]	< 20	< 20	< 20	< 20	< 20
LIT2	30	< 20	< 20	< 20	< 20	< 20	< 20
	60	< 20	< 20	< 20	< 20	< 20	< 20
LMO	30	< 20	30.80 ± 3.02[c,d]	< 20	< 20	< 20	< 20
	60	< 20	43.43 ± 1.21[c]	< 20	< 20	< 20	< 20
LIT3	30	< 20	28.78 ± 2.88[c,d]	< 20	< 20	< 20	< 20
	60	32.66 ± 2.89[c,d]	38.26 ± 4.67[c]	< 20	< 20	< 20	< 20
LHU	30	< 20	21.79 ± 1.18[d]	< 20	< 20	< 20	< 20
	60	23.19 ± 1.12[e]	33.67 ± 1.78[c,d]	< 20	< 20	< 20	< 20
LIT1-H	30	29.37 ± 2.36[d]	25.43 ± 1.32[c,d]	30.12 ±3.14[d]	24.99 ± 1.78[e]	< 20	< 20
	60	42.50 ± 6.24[c,d]	38.80 ± 3.89[c]	49.84 ± 2.22[c]	34.21 ± 1.51[d,e]	< 20	48.34 ± 5.75[c]
LIT2-H	30	38.62 ± 4.35 [c,d]	25.22 ± 1.19[c,d]	47.41 ± 4.74[c]	38.55 ± 1.42[d]	< 20	< 20
	60	38.19 ± 1.28 [c,d]	50.99 ± 3.89[b,c]	63.04 ± 3.73[b]	54.91 ± 4.70[c]	< 20	61.77 ± 8.69[b,c]
LMO-H	30	33.09 ± 1.03 [c,d]	< 20	56.20 ± 3.84[b,c]	32.89 ± 2.02[e]	< 20	< 20
	60	50.39 ± 1.63[c]	23.89 ± 3.18[d]	73.44 ± 1.94[b]	58.33 ± 2.02[c]	28.00 ± 2.19[c]	67.95 ± 1.90[b]
LIT3-H	30	29.18 ± 3.66[d]	< 20	< 20	< 20	< 20	< 20
	60	39.19 ± 2.13[c,d]	< 20	53.61 ± 1.79[b,c]	45.81 ± 1.19[c,d]	< 20	49.57 ± 1.34[b,c]
LHU-H	30	37.61 ± 3.26 [c,d]	< 20	< 20	28.18 ± 1.43[e]	< 20	< 20
	60	46.23 ± 1.52[c]	38.16 ± 2.00[c]	66.97 ± 1.03[b]	62.17 ± 1.28[c]	< 20	58.83 ± 4.72[b,c]
ISL[b]	30	79.29 ± 1.51[b]	83.57 ± 0.75[a]	99.85 ± 1.72[a]	82.02 ± 1.49[b]	79.81 ± 0.67[b]	94.25 ± 0.68[a]
	60	96.58 ± 0.15[a]	95.98 ± 0.41[a]	96.88 ± 0.15[a]	95.39 ± 0.33[a]	92.96 ± 0.70[a]	97.28 ± 0.38[a]
GLY[c]	30	< 20	< 20	< 20	< 20	< 20	< 20
	60	< 20	21.35 ± 1.76[d]	< 20	< 20	< 20	< 20
18β[d]	30	31.63 ± 3.78 [c,d]	< 20	< 20	< 20	< 20	< 20
	60	80.75 ± 2.16[b]	61.59 ± 5.29[b]	94.77 ± 0.46[a]	73.97 ± 2.35[b]	91.03 ± 0.45[a]	94.63 ± 0.22[a]

[a] Mean value from two independent measurements with five parallel wells

[b] isoliquiritigenin

[c] glycyrrhizin

[d] 18β-glycyrrhetinic acid

different letters along columns indicate statistical differences among samples were assessed through Tukey's test ($p < 0.05$).
